# Comparison of tracheal diameter in non-brachycephalic versus brachycephalic dogs using manubrium and thoracic inlet tracheal indexes

**DOI:** 10.3389/fvets.2024.1454930

**Published:** 2024-10-09

**Authors:** Ayman A. Mostafa, Kaitlyn McCutcheon, Clifford R. Berry

**Affiliations:** ^1^Department of Small Animal Surgery and Radiology, Faculty of Veterinary Medicine, Cairo University, Giza, Egypt; ^2^Department of Veterinary Clinical Sciences, College of Veterinary Medicine, Western University of Health Sciences, Pomona, CA, United States; ^3^Diagnostic Imaging, Department of MBS, College of Veterinary Medicine, North Carolina State University, Raleigh, NC, United States

**Keywords:** caudal cervical, intra-thoracic, radiography, tracheal hypoplasia, tracheal lumen diameter

## Abstract

**Introduction:**

Narrowed tracheal lumen diameter (TLD) in dogs caused by congenital hypoplasia or acquired tracheal stenosis can result in adverse health effects. Standardized tracheal scores calculated from radiographic measurements have been used to assess tracheal diameter however comparisons have not been made to characterize differences in tracheal lumen among breeds.

**Methods:**

The main objective of this study was to compare tracheal scores at three regions of the trachea among non-brachycephalic dogs, non-bulldog brachycephalic dogs, and bulldogs. Medical records and thoracic radiographs of clinically normal dogs were reviewed. The TLDs 79 of three different tracheal regions (caudal cervical, thoracic inlet, and intrathoracic) were standardized by the manubrium length (ML) and thoracic inlet distance (Ti-D) to calculate the manubrium and thoracic inlet tracheal indexes (M-TI and Ti-TI) at each region. Statistical analysis was used to analyze the differences in tracheal scores among the three breed populations.

**Results:**

Overall, M-TI and Ti-TI varied significantly (*p* < 0.0001) at the three tracheal levels among the three breed populations. Bulldogs and non-bulldog brachycephalic breeds possessed lower (*p* < 0.016) M-TI and Ti-TI than non-brachycephalic breeds at the three tracheal regions, and bulldogs possessed the lowest M-TI and Ti-TI scores at all measured regions.

**Conclusion:**

Averaged M-TIs <0.38, <0.34, <0.32 in non-brachycephalic, non-bulldog brachycephalic, and bulldog breeds, respectively, may indicate tracheal hypoplasia. Utilizing M-TI can be recommended for the assessment of canine TLD however further investigation in dogs with concurrent respiratory diseases is warranted.

## 1 Introduction

Narrowing of the tracheal lumen in dogs may occur due to acquired tracheal stenosis, tracheal collapse, or congenital tracheal hypoplasia. Congenital tracheal hypoplasia is an anatomical abnormality associated with brachycephalic airway obstructive syndrome, occurring due to opposition or overlapping of cartilage rings in the trachea. This results in a diffusely narrow tracheal lumen (1). Although predominately affecting English Bulldogs, other breeds may also be affected (2–5). Narrowing of the trachea can negatively impact the quality of life due to increased respiratory resistance (6, 7). Furthermore, narrowing may result in respiratory distress, a potentially fatal complication necessitating medical intervention (8). Hence, multiple methodologies involving computed tomography and radiography have been described to assess tracheal diameter for diagnosing tracheal stenosis and hypoplasia, as well as to monitor the progression of tracheal constriction (4, 9–17). Among the various imaging modalities available, radiography predominates as the most common diagnostic modality utilized in clinical practice (13, 18, 19). Conventionally employed radiographic techniques have used the thoracic inlet distance (Ti-D) and the proximal width of rib number three as normalizing parameters for the tracheal lumen diameter (TLD) (1, 4, 7, 9, 12, 20). Recently, the length of the manubrium (ML) has demonstrated a strong correlation to tracheal diameter and has been established as a viable alternative for evaluating tracheal lumen diameters (16, 17). Previous studies have proposed potential reference ranges of standardized tracheal measurements; however, studies have not investigated objective tracheal diameter differences among breed populations at different tracheal levels using the different standardized parameters.

The objective was to compare standardized tracheal lumen diameters (TLDs) to identify differences in lumen diameter among bulldogs, non-bulldog brachycephalic breeds, and non-brachycephalic dogs. Tracheal diameters were measured at three defined regions along the trachea (caudal cervical, thoracic inlet, and intrathoracic, and averaged) and standardized using the length of the manubrium (ML) and thoracic inlet distance (Ti-D). Our hypothesis is that normalized TLD will differ significantly among breed populations, with brachycephalic breeds possessing lower tracheal indexes compared to non-brachycephalic breeds. The results of this study, in combination with previous recent studies conducted by the same authors, are expected to contribute to establishing a radiographic protocol to screen for tracheal hypoplasia in canine patients.

## 2 Methods

### 2.1 Population

Medical records and thoracic radiographs were obtained for small breed dogs admitted to the Small Animal Hospital at the University of Florida, College of Veterinary Medicine from May 2005 to December 2020. Thus, ethical review and approval were not required as medical records and radiographs reviewed were obtained during the administration of routine veterinary care. The study population consisted of three groups of client-owned dogs (non-brachycephalic, non-bulldog brachycephalic, and bulldog breeds) without clinical or radiographic evidence of tracheal, pulmonary, or cardiovascular disease. Dogs were excluded with evidence of an esophageal abnormality, a redundant tracheal membrane, tracheal hypoplasia, or a thickened soft palate. Excluded esophageal abnormalities included neoplasia, megaesophagus, or an esophageal foreign body. Dogs with a history of oral, neck, or chest surgery were excluded as were dogs with evidence of geriatric pulmonary and vascular changes such as mineralization and fibrosis. Radiographs were presumed to have been in animals without sedation and captured at peak inspiration. Animals with radiographic abnormalities of the manubrium (i.e., short, fused, abnormally shaped) were excluded. A short manubrium was previously defined as a length equal to or shorter than the second sternal segment (16, 17). Acceptable manubrium conformations included elongated, bullet-shaped, rectangular, or camel head/neck shaped (21, 22).

### 2.2 Radiographic measurements

**Figure 1:**
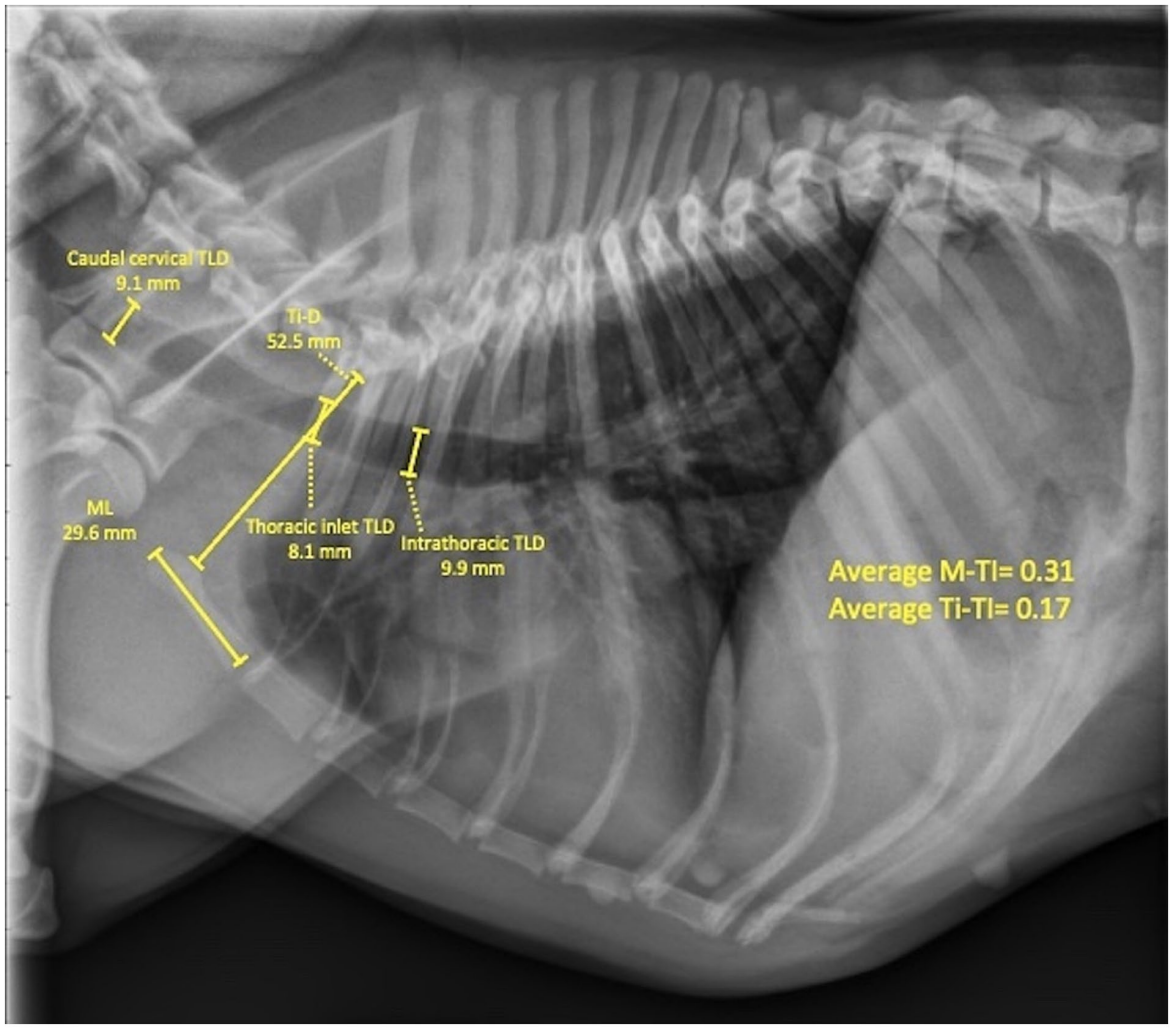
Right lateral thoracic radiographic view of a 9.6-year-old, female (spayed) French bulldog with no clinical or radiographic evidence of respiratory or cardiovascular disease showing measurements of tracheal lumen diameters (TLDs) at caudal cervical, thoracic inlet, and intrathoracic tracheal regions, as well as measuring the manubrium length (ML) and thoracic inlet distance (Ti-D) for calculating the corresponding manubrium (M-TI) and thoracic inlet (Ti-TI) tracheal indexes. Note the associated thoracic vertebral anomaly (TVA).

The quality and positioning of obtained radiographs were approved by a board-certified veterinary radiologist (CRB), and all measurements were completed by a sole investigator on right lateral thoracic views (AAM). Analyzed radiographs were retrieved with an image archiving communication system (Merge PACS, Merge Healthcare Inc., Chicago, Ill) and a medical workstation. Caudal cervical tracheal diameter was measured at the middle of the C5 vertebra, thoracic-inlet tracheal diameter was measured at the level of the caudal C7 vertebra, and intrathoracic tracheal diameter was measured midway between the thoracic inlet and carina, at the level of the mid-T2 to mid-T3 vertebrae (16, 17) (Figure 1). To accommodate for the impact of variations resulting from inter-breed differences, the absolute and averaged tracheal diameter values were normalized by the manubrium length (ML) and thoracic-inlet distance (Ti-D). The ML was measured from the most cranial point of the manubrium to the caudal aspect (16, 17) (Figure 1). The Ti-D was measured as the distance from the cranioventral aspect of the T1 vertebra to the highest point on the craniodorsal aspect of the manubrium (16, 17) Manubrium-tracheal index (M-TI = tracheal lumen diameter/ML) and thoracic inlet-tracheal index (Ti-TI = tracheal lumen diameter/Ti-D) were then calculated at each of the three tracheal regions and compared among three dog populations (non-brachycephalic, non-bulldog brachycephalic, and bulldog breeds).

### 2.3 Statistical analysis

In accordance with the central limit theorem, which states that the distribution of means of large data sets is assumed to be approximately normal, variables were presumed to possess a normal distribution thus parametric tests were conducted for analysis (23). Data is presented as means (±SDs) and 95% confidence intervals were calculated for each measured variable. ANOVA was used to compare variables of interest with statistical significance set to a *p*-value<0.05. Tukey’s test was performed to characterize the difference in means. All data analysis was accomplished using GraphPad Prism software (GraphPad Prism version 8.0.0 for Windows, San Diego, California, USA).

## 3 Results

### 3.1 Populations

A total of 261 non-brachycephalic, non-bulldog brachycephalic, and bulldog breeds met the inclusion criteria. Overall, there were significant differences (*p* < 0.0001) in age and body weight among the populations. Age and body weight did not differ (*p* ≥ 0.06) between non-brachycephalic and non-bulldog brachycephalic breeds (Table 1).

**Table 1:** Means (±SDs) and 95% CIs for the age, body weight, and radiographic measurements of absolute, average, and normalized tracheal lumen diameters at levels A (mid-C5), B (Ca-C7), and C (mid-T2-3) for 261 “healthy” non-brachycephalic, non-bulldog brachycephalic and bulldog breeds.

	Non-brachycephalic (*n* = 81)		Non-bulldog brachycephalic (*n* = 80)		Bulldogs (*n* = 100)		*p*-value (<0.05)	
	Mean ± SD	95% CI	Mean ± SD	95% CI	Mean ± SD	95% CI	ANOVA test	Tukey’s test
Age/y	9.4 ± 3.4	8.6–10.1	8.1 ± 3.9	7.3–9.0	5.6 ± 3.0	5.0–6.2	*p* < 0.0001	0.064*, <0.0001^#^, <0.0001^•^
Body weight/kg	10.2 ± 5.5	8.9–11.5	8.8 ± 6.5	7.3–10.3	23.3 ± 11.2	21.0–25.6	*p* < 0.0001	0.587*, <0.0001^#^, <0.0001^•^
Absolute tracheal luminal diameter/mm (TD)								
Level A (Mid-C5), caudal cervical region	13.4 ± 2.9	12.7–14.1	10.8 ± 3.2	10.1–11.6	12.9 ± 3.7	12.1–13.7	*p* < 0.0001	<0.0001*, 0.570^#^, 0.0004^•^
Level B (Ca-C7), thoracic inlet region	10.6 ± 2.7	10.0–11.2	8.6 ± 3.0	7.9–9.2	11.4 ± 3.3	10.8–12.1	*p* < 0.0001	<0.0001*, 0.151^#^, <0.0001^•^
Level C (Mid-T2-3), midway between thoracic inlet and carina	11.9 ± 3.0	11.2–12.5	9.5 ± 3.3	8.8–10.3	12.8 ± 3.5	12.1–13.5	*p* < 0.0001	<0.0001*, 0.141^#^, <0.0001^•^
Average tracheal luminal diameter	11.9 ± 2.8	11.3–12.6	9.7 ± 3.2	8.9–10.4	12.2 ± 3.3	11.5–12.9	*p* < 0.0001	<0.0001*, 0.888^#^, <0.0001^•^
Normalizing parameters/mm								
Manubrium length (ML)	30.6 ± 7.4	28.9–32.2	27.5 ± 8.4	25.6–29.3	38.2 ± 9.6	36.4–40.1	*p* < 0.0001	0.06*, <0.0001^#^, <0.0001^•^
Thoracic inlet distance (Ti-D)	54.6 ± 10.6	52.3–57.0	48.6 ± 13.6	45.5–51.6	69.4 ± 13.7	66.7–72.1	*p* < 0.0001	0.008*, <0.0001^#^, <0.0001^•^
Manubrium tracheal index (M-TI) = TD/ML								
M-TI, C5 (Level A)	0.45 ± 0.07	0.43–0.46	0.41 ± 0.10	0.39–0.43	0.35 ± 0.06	0.33–0.36	*p* < 0.0001	0.016*, <0.0001^#^, <0.0001^•^
M-TI, C7 (Level B)	0.35 ± 0.06	0.34–0.36	0.32 ± 0.07	0.30–0.33	0.30 ± 0.05	0.29–0.31	*p* < 0.0001	0.002*, <0.0001^#^, 0.195^•^
M-TI, T2-3 (Level C)	0.39 ± 0.06	0.38–0.40	0.35 ± 0.07	0.34–0.37	0.34 ± 0.05	0.33–0.35	*p* < 0.0001	0.0001*, <0.0001^#^, 0.276^•^
Average M-TI	0.40 ± 0.05	0.38–0.41	0.36 ± 0.08	0.34–0.38	0.33 ± 0.05	0.32–0.34	*p* < 0.0001	0.002*, <0.0001^#^, 0.003^•^
Thoracic inlet tracheal index (Ti-TI) = TD/Ti-D								
Ti-TI, C5 (Level A)	0.25 ± 0.04	0.24–0.26	0.23 ± 0.05	0.22–0.24	0.19 ± 0.04	0.18–0.20	*p* < 0.0001	0.003*, <0.0001^#^, <0.0001^•^
Ti-TI, C7 (Level B)	0.19 ± 0.04	0.19–0.20	0.18 ± 0.04	0.17–0.18	0.16 ± 0.03	0.16–0.17	*p* < 0.0001	0.002*, <0.0001^#^, 0.052^•^
Ti-TI, T2-3 (Level C)	0.22 ± 0.04	0.21–0.23	0.20 ± 0.04	0.19–0.20	0.18 ± 0.03	0.18–0.19	*p* < 0.0001	0.0002*, <0.0001^#^, 0.050^•^
Average Ti-TI	0.22 ± 0.04	0.21–0.23	0.20 ± 0.04	0.19–0.21	0.18 ± 0.03	0.17–0.18	*p* < 0.0001	0.0009*, <0.0001^#^, 0.0001^•^

^*^Non-brachycephalic vs. non-bulldog brachycephalic, ^#^Nonbrachycephalic vs. bulldogs, •Non-bulldog brachycephalic vs. bulldogs.

#### 3.1.1 Non-brachycephalic breeds

Medical records and thoracic radiographs of 87 dogs were reviewed for inclusion criteria. Five of the 87 dogs were excluded due to a short manubrium, and one was excluded due to a fused manubrium. A total of 81 dogs were then included in the study. Means age and body weight were 9.4 years and 10.2 kg, respectively (Table 1). Specific breeds represented were: 19 (23.5%) Poodles; 10 (12.3%) each of Jack Russell Terriers and Miniature Schnauzers; 9 (11.1%).

Dachshunds; 7 (8.6%) Beagles; 6 (7.5%) Italian Greyhounds; 4 (5%) each of Cocker Spaniel, Pembroke Welsh Corgi, and Miniature Pinschers; 3 (3.7%) each of Shiba Inu and Chinese Crested; and 2 (2.5%) Scottish Terriers. Forty-three total males were included (37 castrated) and 38 females (35 spayed).

#### 3.1.2 Non-bulldog brachycephalic breeds

Medical records and thoracic radiographs of 88 dogs were reviewed for inclusion. Of the 88 dogs, eight were excluded due to observed manubrium abnormalities; short (4 dogs), fused (3 dogs), and deformed (1 dog) manubriums. A total of 80 dogs then met the inclusion criteria with means age and body weight of 8.1 years and 8.8 kg, respectively (Table 1). Enrolled breeds included 16 Chihuahuas (20%), 11 Boston Terriers (13.7%), 10 each of Pugs, Pekingese, and Cavalier King Charles Spaniels (12.5%), 4 each of Shih Tzus, Pomeranians, and Miniature Shar Peis (5%), 3 Staffordshire Bull Terriers (3.8%), and 2 each of Lhasa Apsos, Bichon Frise, Brussels Griffons, and Chow Chows (2.5%). Forty-four males (29 castrated) and 36 females (33 spayed) were included.

#### 3.1.3 Bulldogs

Medical records and thoracic radiographs of 118 bulldogs were reviewed. Fifteen were excluded due to the presence of a short manubrium and three were excluded due to the presence of fused manubriums. One hundred bulldogs then met the inclusion criteria including 34 French bulldogs, 33 English bulldogs, and 33 American bulldogs. Means age and body weight were 5.8 years and 23.3 kg, respectively (Table 1). Included were 54 males (36 castrated) and 46 females (37 spayed). Thoracic vertebral anomalies (TVA) were identified in 20 English bulldogs, 17 French bulldogs, and one American bulldog (38% total).

### 3.2 Radiographic measurements

**Figure 2:**
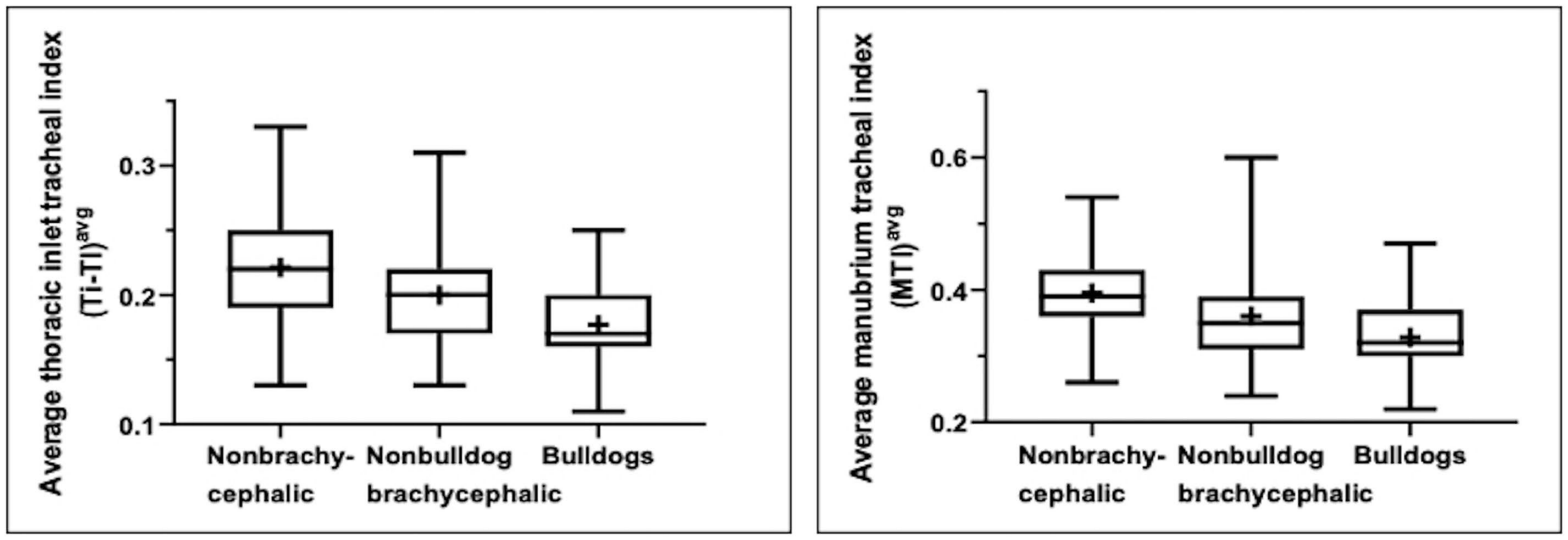
Box-and-whisker plots of the average manubrium tracheal index **(A)** and thoracic inlet tracheal index **(B)** for a total of 261 “normal” non-brachycephalic, non-bulldog brachycephalic, and bulldog breeds. Boxes and whiskers represent the 25th to 75th percentiles and ranges, respectively; crosses represent means and lines represent medians.

Tracheal indexes (M-TI and Ti-TI) varied significantly (*p* < 0.0001) at the three tracheal regions among the three breed populations (Table 1). Bulldogs and non-bulldog brachycephalic breeds possessed lower (*p* ≤ 0.016) M-TI and Ti-TI at the three regions along the trachea compared to non-brachycephalic dogs. There was no difference (*p* ≥ 0.050) in the tracheal indexes between bulldogs and non-bulldog brachycephalic breeds at the thoracic inlet and intrathoracic tracheal lumens; however, the caudal cervical tracheal region was narrower (*p* < 0.0001) in bulldogs (Table 1). Averaged M-TI and Ti-TI values were significantly lower (i.e., narrower tracheal diameter) in bulldogs versus non-bulldog brachycephalic (*p* ≤ 0.003) and non-brachycephalic (*p* < 0.0001) breeds (Table 1; Figure 2). The averaged tracheal indexes (M-TI and Ti-TI) were significantly lower (*p* ≤ 0.002) in non-bulldog brachycephalic compared to non-brachycephalic dogs (Table 1; Figure 2).

## 4 Discussion

The reported results serve as a comparison among breeds regarding tracheal diameters and the radiographic techniques used to standardize measurements of the trachea. Understanding the differences in tracheal diameters among breed populations is crucial for the assessment of tracheal narrowing in canines and for prompt diagnosis and intervention. Additionally, establishing normal reference ranges for canine tracheal diameters may aid in developing a screening protocol that could be beneficial in the diagnosis of tracheal hypoplasia and the selection of appropriate management in affected dogs. Congenital tracheal hypoplasia occurs as a diffuse lesion along the entire length of the trachea, from larynx to carina, with the tracheal lumen uniformly narrowed (7). As such, tracheal hypoplasia cannot be localized to a specific area of the trachea, thus obtaining measurements at multiple tracheal levels is necessary to establish normal tracheal diameters. Furthermore, investigating the usefulness of different methodologies for calculation of tracheal indexes improves the reliability of tracheal measurements obtained on radiographs.

Assessment of tracheal diameter has been accomplished using different imaging modalities and techniques, such as radiography and CT, to monitor and diagnose canine tracheal hypoplasia and tracheal stenosis (4, 9, 11–15). Despite the limitations of radiography and the underestimation of luminal diameter compared to CT, radiography remains the most common diagnostic utilized in the assessment of canine tracheal diameter due to the accessibility and lack of need for sedation (13). While radiographs analyzed for this study were presumed to be taken at peak inspiration and without sedation, the described techniques are applicable to tracheal hypoplasia if these conditions are not met as canines with tracheal hypoplasia do not show changes in TLDs corresponding to the phase of respiration (1). The most prevalent methods of assessing tracheal diameter include the thoracic inlet distance (Ti-TI) or proximal rib three width (PR3-TS) for standardized tracheal measurements, however both Ti-TI and PR3-TS possess questionable value due to the reported poor observer agreement identified impacting the reliability of the results (4). Furthermore, the width of the third proximal pair of ribs did not appear consistent on all lateral thoracic radiographs possibly due to rotation/tilting of the dog during positioning or superimposition of the rib pairs (24). Superimposition of the ribs is of particular concern in dogs with thoracic vertebral anomalies and subsequent crowding of the ribs, as was common among our bulldog population. Due to the potential for error and anatomical variability, proximal rib three was not used as a measurement in this study.

To limit the impact of anatomical variations on Ti-TI, the same landmarks were consistently utilized measuring the thoracic inlet from the cranio-ventral aspect of the first thoracic vertebra to the highest point of the cranial manubrium. Despite this, the Ti-TI technique may still be susceptible to influence from thoracic vertebral anomalies (TVA). In 38% of the bulldogs investigated during this study, a thoracic inlet distance that appeared relatively longer and a caudally displaced thoracic inlet tracheal region were noted due to the presence of a TVA. This may have impacted the results of Ti-TI calculated among bulldogs in this study. Instead, utilizing the M-TI procedure would be recommended particularly in brachycephalic breeds with TVA to objectively evaluate their tracheal diameter and screen for tracheal hypoplasia.

The lowest tracheal scores were found at the thoracic inlet in all breed populations using both M-TI and Ti-TI, with the largest variation in scores existing between the thoracic inlet and the caudal cervical locations. This is consistent with findings in previous studies on canine tracheal diameter that have also indicated a narrower tracheal lumen diameter at the thoracic inlet. The smaller diameter of tracheal rings at the thoracic inlet has been attributed to a change in the direction of the trachea at the thoracic inlet, which is relatively small and surrounded by bones (25). In recent studies, a larger difference was found for the thoracic inlet tracheal diameter compared to the caudal cervical tracheal diameter in non-brachycephalic and non-bulldog brachycephalic breeds (20.9 and 20.4% narrower, respectively) than to the intrathoracic tracheal diameter (10.9% narrower) (16, 17). Interestingly, bulldogs showed less of a difference between thoracic inlet and caudal cervical tracheal lumens compared to non-brachycephalic and brachycephalic breeds. In bulldogs, the difference between the thoracic inlet compared to the caudal cervical and intrathoracic lumen was calculated to be 11.6 and 10.9%, respectively. This may be due to the overall narrower tracheal lumen in bulldogs compared to other breeds, contributing to relatively less narrowing at the thoracic inlet. These are all greater differences than was found in large breed dogs, in which the mean thoracic inlet tracheal diameter was calculated to be 5.7% narrower than the caudal cervical and 7.6% narrower than the intrathoracic (25). Thus, our results indicate that the thoracic inlet tracheal diameter is narrower compared to other tracheal levels in small breeds, brachycephalic breeds, and bulldogs than in large breed dogs.

No significant difference in the calculated Ti-TI or M-TI for the thoracic inlet (*p* = 0.052 and *p* = 0.195, respectively) nor intrathoracic region (*p* = 0.050 and *p* = 0.276, respectively) was found between brachycephalic breeds and bulldogs. However, a significant difference was found at the caudal cervical level between bulldogs and brachycephalic breeds (*p* < 0.0001), bulldogs and non-brachycephalic breeds (*p* < 0.0001), and brachycephalic breeds and non-brachycephalic breeds (*p* = 0.003 for T-TI and *p* = 0.016 for M-TI). These findings suggest that the greatest difference in tracheal diameter among bulldogs and non-bulldog brachycephalic breeds exists at the caudal cervical level and bulldogs have a narrower tracheal lumen at the caudal cervical level than either brachycephalic dogs or non-brachycephalic dogs. Additionally, a significant difference was found in the Ti-TI and M-TI at the thoracic inlet between non-brachycephalic breeds compared to both brachycephalic dogs (*p* = 0.002) and bulldogs (*p* < 0.0001). Non-bulldog brachycephalic breeds and bulldogs had significantly narrower tracheal diameters at the thoracic inlet than other small breed dogs evaluated.

The lowest average TLD using M-TI and Ti-TI was calculated in bulldogs, while the highest average was found in non-brachycephalic breeds. A significant difference (*p* < 0.0001) was found in the average scores for bulldog breeds versus non-brachycephalic and non-bulldog brachycephalic breeds using both M-TI and Ti-TI. Bulldogs possessed the lowest average tracheal indexes, with the average M-TI calculated to be 8.3% lower than in non-bulldog brachycephalic breeds and 17.5% less than in non-brachycephalic small breeds. On the other hand, the average Ti-TI for bulldogs was calculated to be 10% less than non-bulldog brachycephalic breeds and 18.2% less than non-brachycephalic small breeds. Tracheal indexes are lower, and thus the tracheal lumen on average is narrower in brachycephalic breeds as compared to non-brachycephalic breeds with bulldogs having significantly lower tracheal indexes on average than all other studied populations. These results support the conclusion that bulldogs have a relatively narrower tracheal lumen in clinically normal dogs. In the present study, 95% confidence intervals calculated for the average M-TIs of non-brachycephalic small breeds, non-bulldog breeds, and bulldogs establish potential lower values for normal tracheal diameters. Average M-TIs for non-brachycephalic small breeds, non-bulldog breeds, and bulldogs of less than 0.38, 0.34, and 0.32, respectively, may indicate tracheal hypoplasia.

Calculated tracheal scores using Ti-TI at the level of the caudal cervical, thoracic inlet, and intrathoracic regions followed the same patterns as M-TI, as did the average M-TI and Ti-TI calculated for each breed population. In recent similar studies performed on non-brachycephalic small breed dogs and non-bulldog brachycephalic breeds, a strong correlation was found between ML and TLD [*rs* = 0.82 in non-brachycephalic breeds and *rs* = 0.81 in non-bulldog brachycephalic breeds (16, 17)]. In these studies, a strong correlation (*rs* = 0.77 in non-brachycephalic breeds and *rs =* 0.83 in brachycephalic breeds) existed between M-TI and Ti-TI (16, 17). The strong correlation found for M-TI, in addition to the close association to Ti-TI, supports the use of M-TI as an alternative method to assess tracheal diameter in non-brachycephalic small breeds, non-bulldog brachycephalic breeds, and bulldogs (16, 17).

A future assessment of inter- and intra-observer variability is warranted to establish repeatability for the use of M-TI in tracheal hypoplasia screening. Another limitation of the current study is the inclusion of only clinically normal canines. Exclusion of canines with respiratory pathologies limits inclusion of canines with brachycephalic airway syndrome, which are those most likely to be affected by tracheal hypoplasia (2–5). Respiratory pathology may also influence tracheal diameter, such as bronchopneumonia and traumatic or infectious tracheal stenosis (6, 12). Inclusion of canines with respiratory abnormalities may provide insight into the degree of hypoplasia that is tolerated before clinical signs are apparent and allow for improved monitoring of the progression of narrowing. Therefore, future studies are indicated to evaluate tracheal diameter in dyspneic compared to normal brachycephalic and non-brachycephalic breeds and to compare standardized TLD among French, English, and American bulldogs.

## 5 Conclusion

Standardized tracheal diameters varied significantly in all studied breed populations, with the narrowest lumen noted at the level of the thoracic inlet and the widest lumen at the caudal cervical level. M-TI could be a viable alternative to Ti-TI for evaluation of tracheal lumen diameters (TLDs) radiographically, especially in bulldogs. Brachycephalic small breeds possess lower tracheal indexes than non-brachycephalic breeds, with bulldogs possessing scores lower than non-bulldog brachycephalic breeds. Averaged M-TIs for non-brachycephalic small breeds, non-bulldog breeds, and bulldogs of less than 0.38, 0.34, and 0.32, respectively, may indicate tracheal hypoplasia, as was demonstrated by 95% confidence intervals constructed for the average M-TI for the three breed populations. These results may be useful in the development of a screening protocol for canine tracheal hypoplasia. Further investigation is needed to assess TLDs in dyspneic canines and to compare tracheal diameters among the three groups of bulldogs (i.e., French, American, and English).

## Data Availability

The original contributions presented in the study are included in the article/supplementary material, further inquiries can be directed to the corresponding author.
